# Commentary: Diagnostic efficacy of fecal markers combined with alarm symptoms in distinguishing functional and organic abdominal pain in children

**DOI:** 10.3389/fped.2026.1964262

**Published:** 2026-09-17

**Authors:** Toshihiko Kakiuchi

**Affiliations:** Department of Pediatrics, Faculty of Medicine, Saga University, Saga, Japan

**Keywords:** chronic abdominal pain, diagnostic accuracy, fecal calprotectin, functional abdominal pain disorders, functional constipation, verification bias

## 1 Introduction

We read with great interest the study by Liu et al., who evaluated the diagnostic performance of fecal calprotectin (FC), fecal occult blood (OB), alarm symptoms, and blood inflammatory markers in 571 children with chronic abdominal pain. The combination of alarm symptoms and fecal markers showed the highest diagnostic performance for distinguishing organic disease from functional abdominal pain disorders (FAPD), with an area under the receiver operating characteristic curve (AUC) of 0.841, sensitivity of 90.6%, and specificity of 70.3% (1). The development of a minimally invasive strategy that may reduce unnecessary endoscopy in children is clinically important. However, two issues deserve particular consideration: the potential influence of partial verification bias on the reported diagnostic performance and the interpretation of mildly elevated FC as indicative of functional constipation.

## 2 Diagnostic accuracy and partial verification bias

First, although Liu et al. appropriately acknowledge verification bias as a limitation, its implications for the reported diagnostic performance warrant further emphasis (2). Among children ultimately classified as having FAPD, upper and/or lower endoscopy was performed when alarm symptoms were present or FC was ≥60 μg/g, and only 69 of 347 children underwent endoscopic evaluation (1). Thus, alarm symptoms and FC not only formed part of the diagnostic strategies under evaluation but also influenced whether endoscopic verification was performed. This dependence is particularly relevant to Strategy 5, which incorporated alarm symptoms and fecal markers and yielded the highest reported diagnostic performance (AUC 0.841, sensitivity 90.6%, and specificity 70.3%) (1). Accordingly, the acknowledged partial verification bias is not merely a general limitation of the retrospective design but bears directly on the interpretation of the principal diagnostic accuracy estimates.

This issue is particularly relevant because the strategy reported to have the best diagnostic performance (Strategy 5) itself incorporated alarm symptoms and fecal markers. Children without alarm symptoms and with FC <60 μg/g were not routinely subjected to endoscopic verification; therefore, occult organic disease in this group cannot be completely excluded. Importantly, this does not imply that all children with chronic abdominal pain should undergo endoscopy, which would be neither clinically appropriate nor ethically justified. Rather, diagnostic accuracy estimates derived from a verification process influenced by components of the index strategy require cautious interpretation. Prospective validation using a predefined diagnostic pathway, together with adequate longitudinal follow-up of test-negative children, would provide stronger evidence for the proposed strategy before its implementation as a gatekeeper for invasive testing.

The choice of the 60 μg/g threshold adds another layer to this issue. Although Liu et al. selected this cutoff for consistency, pediatric FC concentrations vary with age and assay characteristics, and the ESPGHAN position paper does not establish 60 μg/g as a universal pediatric cutoff (3). Because this same threshold influenced both FC positivity and the decision to perform endoscopic verification, its selection may also affect the resulting diagnostic accuracy estimates.

## 3 Mildly elevated fecal calprotectin and functional constipation

Second, the interpretation of mildly elevated FC as indicative of functional constipation appears stronger than supported by the presented data. In the abstract, Liu et al. conclude that mild FC elevation is likely indicative of functional constipation rather than inflammation (1). However, the subgroup underlying this interpretation is not clearly characterized. The authors report 31 children diagnosed with functional constipation, with an FC positivity rate of 19.4% compared with 13.8% among non-constipated children with FAPD; this difference was not statistically significant (*P* = 0.09) (1). Notably, Table 2 reports constipation in both the organic disease and FAPD groups, making the denominator and diagnostic classification of the functional constipation subgroup difficult to ascertain from the published data. These data therefore show, at most, a non-significant association between constipation status and FC positivity within the reported subgroup and do not establish functional constipation as the cause of FC elevation.

This distinction is also relevant when considering current pediatric guidance. The ESPGHAN position paper on FC states that FC concentrations in most children with functional constipation do not differ from those in healthy controls and concludes that FC has no utility in differentiating functional from organic constipation (3). FC values are also influenced by age, assay characteristics, medications, and other individual factors (3). Therefore, a mildly elevated FC concentration may be better regarded as an indeterminate finding requiring interpretation in the context of the clinical presentation rather than as positive evidence supporting a functional diagnosis. Repeat testing instead of immediate endoscopy may be reasonable in selected children without alarm features, but this pragmatic approach should be distinguished from attributing the FC elevation itself to functional constipation.

## 4 Discussion

The study by Liu et al. highlights the potential value of combining fecal markers with clinical features in the evaluation of pediatric chronic abdominal pain. Nevertheless, before such a strategy is incorporated into routine clinical practice, its diagnostic performance should be prospectively validated using methods that minimize test-dependent verification. Equally importantly, the interpretation of mildly elevated FC should remain proportional to the supporting evidence. A non-invasive diagnostic strategy is most useful when it reduces unnecessary procedures without introducing unwarranted diagnostic certainty.
